# Protein Hydrolysates from Brewing By-Products as Natural Alternatives to ACE-Inhibitory Drugs for Hypertension Management

**DOI:** 10.3390/life12101554

**Published:** 2022-10-07

**Authors:** Rita Ribeiro-Oliveira, Zita E. Martins, Miguel Ângelo Faria, Joana Beatriz Sousa, Isabel M. P. L. V. O. Ferreira, Carmen Diniz

**Affiliations:** 1 LAQV/REQUIMTE, Laboratory of Pharmacology, Department of Drug Sciences, Faculty of Pharmacy, University of Porto, 4050-313 Porto, Portugal; 2 LAQV/REQUIMTE, Laboratory of Bromatology and Hydrology, Department of Chemical Sciences, Faculty of Pharmacy, University of Porto, 4050-313 Porto, Portugal

**Keywords:** hypertension, cardiovascular diseases, bioactive peptides, brewing by-products, brewer’s spent grain, brewer’s spent yeast

## Abstract

**Simple Summary:**

Hypertension is the predominant risk factor for cardiovascular disease, which is the leading cause of mortality and morbidity worldwide. The search for natural compounds with antihypertensive properties, such as bioactive peptides from brewing by-products (spent grain and yeast), which are less likely to cause severe side effects compared with anti-hypertensive drugs, is of major importance to reduce cardiovascular events. Since oral intake of these peptides may modify their expected effects, the aim of the present study was to simulate oral administration and evaluate the impact of gastrointestinal digestion, intestinal absorption, and liver metabolism on the effectiveness of those bioactive peptides and determine their potential to be used as supplements or nutraceuticals as well as anti-hypertensive drugs before moving forward to animal studies. Results showed that peptides derived from the brewing industry maintain or present higher antihypertensive activity after simulation of oral administration, validating the usefulness of these peptides to reduce the risk, ameliorate, or treat primary hypertension. In conclusion, this study reinforces, through in vitro studies, the benefits of oral administrated brewing bioactive peptides to directly manage hypertension by lowering blood pressure, thus being promising compounds.

**Abstract:**

The treatment of hypertension is of major importance to reduce the risk of cardiovascular disease, the leading cause of death worldwide. Angiotensin-converting enzyme (ACE) inhibitors are anti-hypertensive drugs associated with several side effects. Natural products, namely bioactive peptides from brewing by-products, brewers’ spent grain (BSG), and yeast (BSY), are promising alternatives since they can inhibit ACE in vitro. However, the oral intake of these peptides may modify their expected inhibitory effect owing to possible changes in active peptides’ bioavailability, which have not been assessed so far. The goal of this study was to simulate oral administration to evaluate BSG/BSY peptides’ effectiveness by submitting protein hydrolysates sequentially to simulated gastrointestinal digestion, intestinal absorption (Caco-2 cells), and liver metabolism (HepG2 cells). MTT assay was used to assess BSG/BSY protein hydrolysates safeness. The ACE-inhibitory potential of initial and final protein hydrolysates (BSY, BSG, and a new product, MIX) were tested using a fluorometric assay and compared with captopril (1 µM, an ACE-inhibitory drug). Simulation of oral administration greatly increased BSY and MIX protein hydrolysates’ ACE-inhibitory capacity, though final MIX and BSG revealed greater ACE-inhibitory potential than captopril. Notwithstanding, all final protein hydrolysates presented ACE-inhibitory capacity, thus being promising compounds to manage hypertension.

## 1. Introduction

Despite all efforts, cardiovascular diseases (CVD) are still the leading causes of mortality and morbidity worldwide [1]. Hypertension (chronic elevation of blood pressure–BP) is often called ‘the silent killer’ because it is a worldwide epidemic that shows no early symptoms and, simultaneously, is the predominant risk factor for CVDs, highly contributing to premature death and disability. Therefore, new preventive strategies are urgently needed to reduce BP in line with one of the global targets for non-communicable diseases: to reduce the prevalence of hypertension by 33% until 2030 [1].

The majority (90–95%) of patients suffer from essential or primary hypertension presenting structural and functional vascular changes largely caused by an overactivation of the renin-angiotensin system (RAS) [2]. The classical RAS axis begins with renin that cleaves angiotensinogen to angiotensin (Ang) I which, in turn, *via* angiotensin-converting enzyme (ACE), generates Ang II, an endogenous active peptide that promotes vasoconstriction, increases oxidative stress, fibrosis, and inflammation [3]. To reverse this scenario, several drugs targeting ACE are currently used in the clinical practice despite of being associated with significant side effects [4], such as captopril, the first ACE-inhibitory drug available on the market [5] and still applied in this research field as a reference compound. Hence, there is an emergent search for natural compounds with antihypertensive properties that are less likely to cause severe side effects while maintaining the therapeutic efficacy. Accordingly, food-derived peptides can also lower BP (inhibiting ACE) and be used as preventive agents [6] as an initial treatment in mild hypertensive patients or as an additional treatment to anti-hypertensive drugs. The search for new food-derived peptides with beneficial health properties is thus relevant and considerable interest has been devoted to the production of such peptides from low-quality food and underutilized and/or wasted materials to promote a circular economy [7]. The brewing industry by-products are among the by-products with the lowest valorization and are available at low or no cost [8,9]. The major brewing by-products, brewers’ spent grain (BSG—the insoluble part of the barley grain) and yeast (BSY—*Saccharomyces cerevisiae*), are rich in proteins that can be used to obtain natural peptides presenting multiple biological activities, including anti-hypertensive activity [7]. Despite this knowledge, the commercial application of these peptides has been delayed, among other reasons, because the confirmation that peptides maintain their bioactive properties after gastrointestinal digestion, intestinal absorption, and liver metabolism is still to be performed [10]. Therefore, we postulated that oral administration could modify the inhibitory effects of protein hydrolysates towards ACE, inactivating or enhancing their bioactive capabilities and thus conditioning the therapeutic outcome. The current study aimed to address this problem by evaluating the impact of simulated gastrointestinal digestion, intestinal absorption, and liver metabolism on the effectiveness of BSG/BSY peptides as ACE inhibitors, while simultaneously assessing their safeness. Additionally, this work also intended to assess the bioactivity of a new protein hydrolysate (MIX) and to select the brewing protein hydrolysate with higher antihypertensive potential to be pursued and possibly marked as a putative supplement, nutraceutical, or even as an anti-hypertensive drug.

## 2. Materials and Methods

### 2.1. Chemicals and Reagents

Alcalase^®^ 2.4L (126741), bile bovine (B3883), captopril (C4042), DMEM-hg medium (D5648), Fluorometric ACE activity assay kit (CS0002), pancreatin (PT545), Penicillin-Streptomycin (P4333), pepsine (P7012), and trypsin-EDTA 0.25% (T4549) were purchased from Sigma (Sintra, Portugal). MEM Non-Essential Amino Acids (X0557) and Glutamine (X0551) were from Biowest (Nuaillé, France). FBS (10270-106) from Gibco, a BCA protein assay kit (23225) from Thermo Fisher Scientific (Waltham, MA, USA), and MTT (Thiazolyl blue tetrazolium bromide; ET1585) from Carbosynth (cymit química S.L., Barcelona, Spain) were used.

### 2.2. Production of Proten Hydrolysates from Brewing By-Products

Brewing by-products (BSY and BSG) were kindly supplied by Super Bock group (Matosinhos, Portugal). BSY from Lager beer (*Saccharomyces pastorianus*) with 2–4 repitchings in the brewing fermentation step was obtained from two different crops (9 April 2021 and 14 September 2021). The BSY samples were supplied as slurry and transported under refrigerated conditions and stored at 4 °C until analysis. BSG samples (15 October 2020) from Super Bock classic brewing process were vacuum-packed and stored in propylene bags at −20 °C until analysis.

#### 2.2.1. BSY Hydrolysates

To breakdown the yeast cell wall, the mechanic disruption process described by Vieira et al. [11] was adopted with some modifications. First, 30–35 g of BSY was distributed by 50 mL falcons, centrifuged at 5000× *g* for 15 min at 4 °C, and washed 3x with 0.1 M sodium phosphate buffer pH 7, alternating centrifuges and resuspensions to remove beer liquor. After the last centrifugation, total nitrogen of approximately 1 g of pellet sample was analyzed by the Kjeldahl method [12] and protein content estimated using the standard nitrogen-to-protein conversion factor of 6.25. The pellet was resuspended 1:1 (*w*:*v*) in 0.1 M sodium phosphate buffer pH 6. BSY resuspension was mixed 1:1 (*v*:*w*) with glass beads with a diameter of 0.22–0.50 mm (22.222.0002, Retsch) in 15 mL precellys flasks. Cell lysis was performed in Precellys^®^ Evolution (Bertin Technologies) with 3 cycles of 30 s at 7500 rpm followed by 1 min in ice. The homogenate was collected and centrifuged at 15,000× *g* for 15 min at 4 °C. The resulting clear supernatant was freeze-dried (Telstar Cryodos-80, Lisboa, Portugaln). Glass beads were separated from the pellet by resuspension in water and decanted (beads are denser), then washed several times and dried in the incubator at 60 °C for future reuse.

All freeze-dried protein-rich isolates were combined and resuspended in 0.1 M sodium phosphate buffer pH 6 to 75% initial volume, agitating for 30–40 min at 4 °C. Protein quantification (diluted 100×) was performed in triplicates using the BCA Protein assay kit and proteases activity (diluted 5× and 10×) was carried out in triplicates according to Cupp-Enyard [13] using a commercial protease (Alcalase) as positive control. Protease activity was expressed as the number of protease units per mL of enzyme (U/mL). One unit of protease activity (U) was defined as the amount of the enzyme needed to catalyze the formation of 1 μmoles of tyrosine per 1 min, at 37 °C and pH 7.5.

Hydrolysis was performed as previously reported by our research group [14]. Briefly, 10 mg/mL of BSY protein (proteases activity = 0.295 U/mL) was incubated at 36 °C for 6 h in agitation; at the end, the solution was heated at 90 °C for 15 min to assure the enzymes inactivation and centrifuged at 10,000× *g* for 10 min. The resulting supernatant containing the protein hydrolysate was freeze-dried and combined.

#### 2.2.2. BSG Hydrolysates

The initial protein content of BSG was estimated by the Kjeldahl method as described above. BSG proteins were extracted according to our previous work [15] with an alkaline solution of 0.5 M NaOH followed by acidic precipitation of protein with a HCl solution. After centrifugation (15,000× *g* for 15 min at 4 °C) the pellet was freeze-dried (Telstar Cryodos-80, Spain) and dissolved 1 g/10 mL in Tris-HCl buffer pH 8 by shaking for 15 min at 4 °C, adjusting the pH to 8, and further shaking for 45 min. At the end, the extract was centrifuged (12,000× *g* for 30 min at 4 °C) and the supernatant containing BSG proteins combined and saved.

The protein content of the supernatant was determined by BCA protein assay. Protein hydrolysis was performed according to Vieira et al. [16] using BSY proteases obtained as described above. Hydrolysates were centrifuged (3000× *g* for 10 min at 4 °C) to separate the undigested substrate (pellet) and to collect the hydrolysate material (supernatant) that was freeze-dried and combined.

#### 2.2.3. MIX Hydrolysates

BSG protein hydrolysates and BSY protein hydrolysates were mixed 50:50 to produce a new product coded as MIX.

### 2.3. Simulation of Oral Administration

#### 2.3.1. Simulated Gastrointestinal Digestion (SGID)

SGID of protein hydrolysates was performed adapting the standardized protocol of INFOGEST [17]. Five g of each freeze-dried sample (BSY, BSG and a 50:50 mixture of BSY:BSG—MIX) were subjected to sequential oral, gastric, and intestinal digestion. Due to the powder consistency of samples, simulated mastication was performed by vortexing. Additionally, in the oral phase, salivary amylase was omitted since the samples did not include starch. Pepsine 2912 U/mg was used in the gastric phase, but no gastric lipase was added, providing the non-fat composition of protein hydrolysates. For the intestinal phase, pancreatin 6 U/mg and bile bovine 1.496 mmol/g were utilized. At the end of this phase, samples were immediately centrifuged at 3000× *g* for 5 min and the resultant supernatant incubated at 95 °C for 5 min to ensure enzymes inactivation. At the end of the procedure, a final volume of 35 mL of digested sample/falcon was obtained, corresponding to 142.86 mg protein hydrolysates/mL. Digests were stored at −20 °C until analysis.

#### 2.3.2. Simulated Intestinal Absorption and Liver Metabolism

Simulated intestinal absorption (Caco-2 cells) and liver metabolism (HepG2 cells) were performed simultaneously, adapting protocols from the literature [18,19]. Briefly, Caco-2 cells were seeded in 6-well plate inserts, while HepG2 cells were seeded in the bottom of the plates, thus, creating a static simple double-layered system of Caco-2 and HepG2 cells separated by a semipermeable membrane culture insert. The samples were added at the top of Caco-2 cells, transported into the HepG2 cells compartment, and, after simulated absorption and metabolism, the final products were collected from the HepG2 cells compartment. The precise conditions used are described below.

Caco-2 cells (Human Caucasian colon adenocarcinoma, from the European Collection of Authenticated Cell Culture) were maintained at 37 °C under a humidified atmosphere of 95% air and 5% CO_2_ in DMEM-hg medium supplemented with 10% FBS, 1% Penicillin-Streptomycin, 1% MEM Non-Essential Amino Acids and 1% Glutamine in T25 flasks. When confluent, cells were washed with PBS (204.14 mg/L KH_2_PO_4_, 765.36 mg/L Na_2_HPO_4_·2H_2_O, 201.31 mg/L KCl, 8766 mg/L NaCl, pH 7.4) and detached with a Trypsin-EDTA 0.25% solution incubating for 7 min at 37 °C. Complete medium was used to inactivate the trypsin, and cells were split 1:20 to new flasks. Caco-2 cells from passages 73–86 were used for the experiments.

HepG2 cells (Human hepatoblastoma, from DSMZ, German Collection) were maintained in DMEM-hg medium supplemented with 10% FBS and 1% Penicillin-Streptomycin in T25 flasks, and kept in the same conditions as Caco-2 cells, except for the incubation time with Trypsin-EDTA 0.25% (10 min). HepG2 cells from passages 11–21 were used for the experiments.

For the absorption assay, Caco-2 cells were passed to T75 flasks and, when ~80% confluent, harvested and 1 × 10^5^ cells/cm^2^ seeded in 6-well plate inserts (734-2720 VWR) with 4.78 cm^2^ of culture area and 0.4 µm pores, as described by Choi et al. [18]. Cells were allowed to differentiate for 21 days in order to display many functions of the small intestinal villus epithelium [19]. Before the experiment, Caco-2 cells were washed with medium without supplements and stabilized for 30 min at 37 °C. Then, the integrity of the Caco-2 monolayers was checked by transepithelial electrical resistance (TEER) using an Epithelial Volt-Ohm-Meter Millicell^®^ ERS-2 (MERS00002, Millipore-Sigma Aldrich, Sintra, Portugal) + Electrodes Millicell^®^ (MERSSTX01, Millipore - Sigma Aldrich, Sintra, Portugal). Cell monolayers with TEER values below 1584 Ω cm^2^ were discarded [19].

Three days before the experiment, HepG2 cells were harvested and 1 × 10^5^ cells/cm^2^ were seeded in a 6-well plate. Before the experiment, cells were washed with medium without supplements and the inserts containing the Caco-2 cells passed to the HepG2 plates for the co-culture system [18]. Then, 1.5 mL of SGID protein hydrolysates diluted 100× in medium without supplements (final concentration of 1.43 mg/mL) were added to the apical side of the insert, while 2.5 mL of fresh medium was added to the basolateral side. The absorption occurred for 120 min at 37 °C; after that, the inserts were removed and the HepG2 plates incubated for more 24 h for the metabolism simulation.

After the absorption simulation, the inserts with Caco-2 cells were washed with medium without supplements and re-used after 30 min stabilization after assuring that TEER values continued above 165 Ω cm^2^, with a maximum of 3 experiments per day per insert. The inserts were also reused after 2 days of incubation in complete medium, which is the time required to ensure the reestablishment of the cell monolayer integrity according to Pires et al. [20]. This protocol was followed from day 21–30 of Caco-2 cells, while they maintain morphofunctional properties [20].

The final product that passed through the Caco-2 monolayer and metabolized for 24 h (with a theoretical concentration of 0.86 mg/mL) was collected and stored at −20 °C until further analysis.

### 2.4. Cytotoxicity Assay

Caco-2 and HepG2 cells’ viability was assessed with the MTT assay as previously reported by Vieira et al. [16] with some modifications. Briefly, 1 × 10^5^ cells/cm^2^ were seeded in a 96-well plate and incubated for 2 days. After that, cells were exposed to various concentrations of BSG, BSY, and MIX protein hydrolysates (0.86, 5, 10, and 20 mg/mL) for 24 h at 37 °C under a humidified atmosphere of 95% air and 5% CO_2_. A negative control (complete medium) and a positive control (cells treated with 0.1% triton X-100 in complete medium) were additionally included in the plate. The medium was aspirated and 100 μL of a 0.4 mg/mL MTT solution (prepared from a 2.5 mg/mL stock solution in PBS) in complete medium was added. The plate was incubated at 37 °C for 30 min (HepG2 cells) or 90 min (Caco-2 cells) protected from light. Then, the MTT solution was aspirated and 100 μL of DMSO added and left for 30 min with agitation and protected from light for the formazan crystals to dissolve. At the end, the absorbance was measured at 570 nm (Synergy HT, Biotek Instruments, Santa Clara, CA, USA) and cell viability (%) was calculated according to the following formula: Absorbance of sample/Absorbance of negative control × 100.

### 2.5. ACE-Inibitory Assay

A fluorometric ACE activity assay kit was used to evaluate the ACE-inhibitory potential of protein hydrolysates before and after simulated oral administration. Initial samples were diluted in DMEM-hg medium without supplements in concentrations theoretically identical to the final products after simulated oral administration (0.86 mg/mL). Captopril 1 µM [21], a clinical antihypertensive drug used to inhibit ACE, was used as an ACE-inhibitory control and dissolved in the same solutions as the initial/final products. ACE from the kit was used as a positive control and diluted in the same solutions as the initial/final products. ACE from the kit was added to the samples, followed by assay substrate and the fluorescence was immediately read (λ_ex_ 320 nm/λ_em_ 405 nm) in kinetic mode in 5 cycles for 5 min in Cytation^TM^ 3 Automated Fluorescence Microscopy (Biotek Instruments, Santa Clata, CA, USA). Samples and control were evaluated in duplicate. A standard curve (RFU vs. nmol) and sample kinetic curve (RFU vs min) were constructed to determine the linear regression equation. Sample enzymatic activity was calculated according as follows:

(1) $$\mathrm{ACE}\;\mathrm{activity}\;\left(\frac{\mathrm{nmol}}{\min}=\mathrm{units}\right)=\frac{{\mathrm{slope}}_{\mathrm{sample}}}{{\mathrm{slope}}_{\mathrm{standard}}}\times \mathrm{dilution}\;\mathrm{factor}$$

ACE activity was expressed as mU wherein one unit of ACE is defined as the amount of enzyme that releases 1 nmol of fluorescent product from the substrate, in 1 min, under the assay conditions, at 37 °C.

The comparison against captopril was calculated as:

(2) $$\mathrm{ACE}\;\mathrm{activity}\;\left(\%\;\mathrm{captopril}\right)=\frac{{\mathrm{ACE}\;\mathrm{activity}}_{\mathrm{sample}}}{{\mathrm{ACE}\;\mathrm{activity}}_{\mathrm{captopril}}}\times 100$$

### 2.6. Statistical Analysis

Statistical analysis was performed with GraphPad Prism (version 8.3, GraphPad Software, La Jolla, CA, USA). The normality of the variables was evaluated with the Kolmogorov–Smirnov test. Since the variables followed a normal distribution, parametric tests were used. Data are presented as mean ± standard deviation (SD) values from n independent experiments. Differences of means were compared using one-way analysis of variance (ANOVA) followed by the Tukey’s or Dunnett’s multiple comparisons *t*-test. A *p*-value lower than 0.05 was considered to denote statistically significant differences.

## 3. Results

### 3.1. Brewing Protein Hydrolysates

The BSY by-product used in the present study contained 33.47 ± 2.46% protein/dry weight. After cell lysis, 55.05 mg/mL of protein was recovered, and the activity of proteases was 1.564 U/mL. The BSG by-product comprised 26.90 ± 1.51% protein/dry weight, resulting in 15.34 mg/mL of protein extracted. These protein-rich extracts were hydrolyzed to obtain protein hydrolysates (containing a mixture of peptides) denominated BSG, BSY, and MIX protein hydrolysate (please see Section 2.2).

### 3.2. Impact of Oral Administration on the ACE-Inhibitory Capability of Brewing Protein Hydrolysates

**Figure 1:**
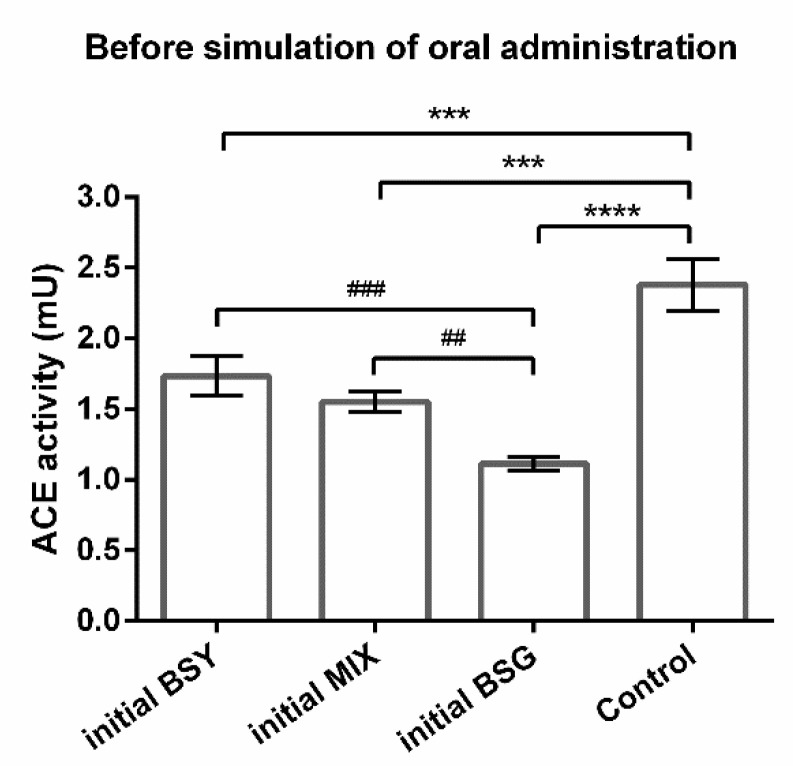
Impact of natural peptides derived from brewing by-products on ACE activity. Effect of 0.87 mg/mL of initial (before simulation of oral administration) brewer’s spent grain (BSG), brewer’s spent yeast (BSY), and 50:50 mixture of BSY:BSG (MIX) protein hydrolysates on angiotensin-converting enzyme (ACE). ACE without treatment was used a control. Values are mean ± SD from 3 independent experiments (duplicates) in each group. Significant differences of ACE activity from control: *** *p* < 0.001 and **** *p* < 0.0001 (One-way ANOVA followed by post hoc Dunnett’s multiple comparisons *t*-test). Significant differences of ACE activity were found between groups: ## *p* < 0.01 and ### *p* < 0.001 (One-way ANOVA followed by post hoc Tukey’s multiple comparisons *t*-test).

To access if the oral route changes the effectivity of the samples, the ACE-inhibitory capacity of brewing protein hydrolysates was evaluated before and after applying a novel protocol of in vitro simulated oral administration. The ACE-inhibitory potential of the initial and final products (before and after simulation of oral administration, respectively), was inferred by comparison with a control (ACE without treatment). Results indicate that 0.86 mg/mL of initial BSG and initial BSY protein hydrolysates can inhibit ACE since they caused a reduction on ACE activity compared with the control (Figure 1). Moreover, the new compound tested, the mixture of both brewing protein hydrolysates (MIX), also demonstrated the potential to inhibit ACE (Figure 1), although the effect was more pronounced for individual BSG protein hydrolysate (Figure 1).

**Figure 2:**
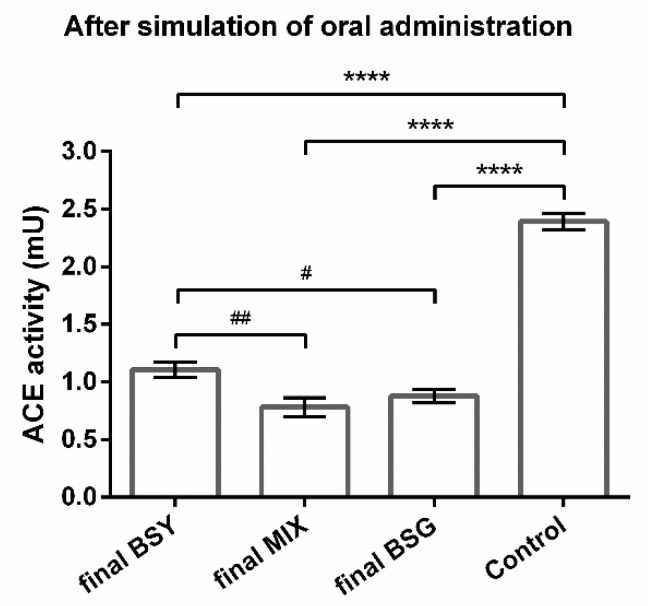
Impact of natural peptides derived from brewing by-products after simulation of oral administration on ACE activity. Effect of final (after simulation of oral administration) brewer’s spent grain (BSG), brewer’s spent yeast (BSY), and 50:50 mixture of BSY:BSG (MIX) protein hydrolysates on angiotensin-converting enzyme (ACE). Protein hydrolysates theoretical concentration of 0.86 mg/mL assuming all hydrolysates were absorbed by Caco-2 cells. ACE without treatment was used a control. Values are mean ± SD from three independent experiments (duplicates) in each group. Significant differences of ACE activity from control: **** *p* < 0.0001 (One-way ANOVA followed by post hoc Dunnetty’s multiple comparisons *t*-test). Significant differences of ACE activity were found between groups: # *p* < 0.05 and ## *p* < 0.01 (One-way ANOVA followed by post hoc Tukey’s multiple comparisons *t*-test).

After simulated oral administration, the final BSG protein hydrolysate maintained its ACE-inhibitory capacity since the effect on enzyme’s activity against the control (Figure 2) remained with the same statistical difference compared to the initial hydrolysates (Figure 1). On the other hand, the final BSY protein hydrolysate greatly improved its ACE-inhibitory capability since the effect on the enzyme’s activity against the control (Figure 2) was more pronounced compared with the initial hydrolysates (Figure 1). Interestingly, the final MIX protein hydrolysate also improved its effect, equaling the final BSG peptides (Figure 2), thus displaying an unexpected potential. The results of BSY and MIX protein hydrolysates indicated that these bioactive peptides are positively influenced by oral administration.

### 3.3. Effectivity of Brewing Protein Hydrolysates as Potential Antihypertensive Compounds

To better understand the potential benefit of brewing peptides on hypertension, their impact on ACE activity was compared with the effect elicited by captopril (at 1 µM dissolved in the same buffer as samples), a conventional anti-hypertensive drug that present ACE-inhibitory capacity. Table 1 presents the impact of initial and final BSG, BSY, and MIX protein hydrolysates expressed as a percentage against captopril. Initial BSY protein hydrolysate induced a 26.8% higher ACE activity regarding captopril, revealing a lower ACE-inhibitory capacity (Table 1). However, after oral administration the effect of BSY peptides and captopril was statistically identical (Table 1). By contrast, initial BSG peptides were more effective in decreasing ACE activity than captopril, a quality that slightly increased after oral administration (Table 1). Surprisingly, initial MIX protein hydrolysate presented a statistically equal potency as captopril, and its ACE-inhibitory capability greatly improved after oral administration (Table 1). Indeed, the changes induced by oral administration on the new product MIX were advantageous, and the final product became approximately 30% more efficient than the clinically used antihypertensive drug captopril (Table 1).

Summing up, contrary to what was observed in the initial protein hydrolysates, all the final brewing protein hydrolysates were as good or more effective than captopril (1 µM), reinforcing the abovementioned results suggesting that the brewing peptides suffer changes during oral administration.

### 3.4. Cytotoxicity Evaluation of Brewing Protein Hydrolysates

**Figure 3:**
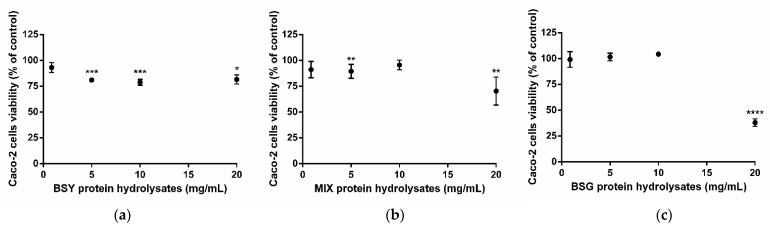
Caco-2 cell viability after incubation with brewing protein hydrolysates. Cells were exposed to (**a**) brewer’s spent yeast—BSY; (**b**) 50:50 mixture of BSY:BSG—MIX; (**c**) brewer’s spent grain—BSG, for 24 h. Results are presented as % of control (cells without treatment) and mean ± standard deviation (SD) values of three independent experiments. Significant differences of cells viability from control: * *p* < 0.05, ** *p* < 0.01, *** *p* < 0.001 and **** *p* < 0.0001 (One-way ANOVA followed by post hoc Dunnett’s multiple comparisons *t*-test).

The viability of Caco-2 cells exposed to 24 h incubation with increasing concentrations of BSY, BSG, and MIX (50:50 mixture) was assessed using the MTT assay. Results are depicted in Figure 3: exposure to 0.8 mg/mL of all the peptides’ samples tested preserved cells viability close to 100% (no significant statistical differences were found compared with control cells—without treatment), while higher concentrations reduced Caco-2 cells viability up to 19% (Figure 3a), 30% (Figure 3b), or by 62% (Figure 3c) in the presence of BSY, MIX, or BSG protein hydrolysates, respectively.

**Figure 4:**
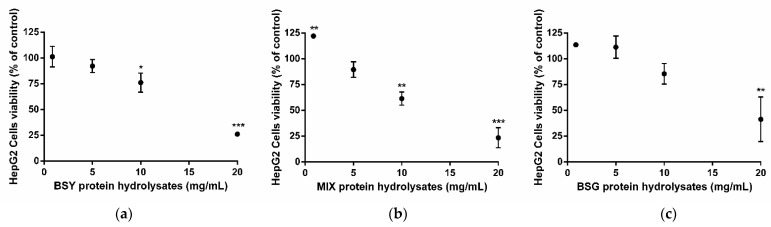
HepG2 cell viability after incubation with brewing protein hydrolysates. Cells were exposed to (**a**) brewer’s spent yeast—BSY; (**b**) 50:50 mixture of BSY:BSG—MIX; (**c**) brewer’s spent grain—BSG, for 24 h. Results are presented as % of control (cells without treatment) and mean ± standard deviation (SD) values of three independent experiments. Significant differences of cells’ viability from control: * *p* < 0.05, ** *p* < 0.01 and *** *p* < 0.001 (One-way ANOVA followed by post hoc Dunnett’s multiple comparisons *t*-test).

In another set of experiments, we evaluated the viability of HepG2 cells after 24 h exposition to BSY, MIX, or BSG protein hydrolysates (Figure 4). At low concentrations (0.86 and 5 mg/mL), all protein hydrolysates preserved, or even increased cell viability compared with control (cells without treatment). By contrast, higher concentrations caused a reduction of cell viability in a concentration-dependent manner for BSY, MIX, or BSG protein hydrolysates, which had induced cell death up to 74%, 77%, or 59%, respectively.

Thus, based on Caco-2 and HepG2 cells’ results, the concentration of 0.86 mg/mL was chosen as the most adequate to pursue and accomplish the proposed goals.

## 4. Discussion

The current study demonstrated, for the first time, the impact of oral administration on the bioactivity of natural peptides derived from the brewing industry, revealing an increase in the ACE-inhibitory capacity of protein hydrolysates, reinforcing the importance of exploring in vitro the influence of the oral route before moving forward to in vivo studies. Additionally, the new brewing protein hydrolysate (MIX) tested exhibited an exceptional efficiency in inhibiting ACE, revealing a 30% greater effect than captopril (Table 1). Moreover, this study corroborated the usefulness of brewing peptides to reduce the risk, ameliorate, or treat primary hypertension since the final products presented similar or higher ACE-inhibitory capacity than captopril, a clinically used antihypertensive drug. Considering the present results, this study suggests BSG and MIX protein hydrolysates as the best brewing products to pursue in order to develop anti-hypertensive products that could be marked as food ingredients, supplements, nutraceuticals, or even drugs with the indication to reduce BP.

The present study reused by-products from the brewing industry (BSG and BSY) to extract proteins and convert them into bioactive peptides with the ability to inhibit ACE in vitro. The protein content of BSG and BSY by-products varies according to the raw ingredients used and brewing conditions that differ among breweries. In our study, the BSG used contained 26.90 ± 1.51% protein/dry weight, which is in accordance with previous works that report 19-30 % dry weight [22]. Meanwhile, the BSY by-product utilized exhibited 33.47 ± 2.46% protein/dry weight, representing a slight lower protein content than literature (35–60% dry weight [23]). The initial BSY and BSG protein hydrolysates obtained demonstrated an ACE-inhibitory capacity (Figure 1) at safe concentrations (Figure 3 and Figure 4), implying a hypotensive potential of brewing protein hydrolysates, which is in line with data reported previously [14,24,25,26,27,28,29,30,31]. Indeed, an in vitro ACE-inhibitory potential ascribed to BSY [14,24,25,26,27] and to BSG [28,29,30] protein hydrolysates/bioactive peptides were reported. To our knowledge, the current study is the first work studying a mixture of both protein hydrolysates (MIX), revealing a relevant ACE-inhibitory capacity (Figure 1b). The concentration of protein hydrolysates used (0.86 mg/mL) in the present work was established after evaluation of potential cytotoxicity using two different cell lines: Caco-2 (to determine the peptides’ impact in cells that constitute the intestinal barrier [32]) and HepG2 cells (to evaluate the peptides’ toxicity [33]). At this low concentration, all peptides were demonstrated to be safe, evidenced by the preservation of Caco-2 (Figure 3) and HepG2 (Figure 4) cell viability. Interestingly, the HepG2 cells exposed to the novel MIX protein hydrolysate exhibited an increase in cell viability (Figure 3b), which might reflect higher metabolic competence. Such increase in cell viability induced by brewing peptides is in accordance with a previous study [27].

The in vitro ACE-inhibitory capability of brewing protein hydrolysates makes them promising natural compounds to manage hypertension. To reach that market, the oral route is the preferred via of administration on a daily basis. Thus, the impact of gastrointestinal events and the bioavailability after intestinal absorption as well as liver metabolism is of vital importance and should be taken into consideration to identify promising bioactive peptides [10]. Some of the prementioned BSY/BSG studies simulated gastro-intestinal digestion [26,27,30], only one of them considered intestinal absorption [27], and none of them studied liver metabolism. The lack of studies considering these aspects is not specific to brewing peptides, but common in other food-derived peptides [34], which is a concern since food-derived antihypertensive peptides are the most studied to date, as previously reported in a recent review [35]. The present study brings, therefore, new insights in the understanding of BSG and BSY peptides’ bioactivity by simulating oral administration. For that purpose, we used a well-established protocol to mimic gastro-intestinal digestion [17] followed by intestinal absorption using the Caco-2 cell line, a widely accepted model that mimics essential aspects of intestinal epithelial barrier [32]. The safe concentration used (0.86 mg/mL) maintained the cells’ integrity that was also confirmed through TEER values, therefore reflecting an intact intestinal barrier ensuring an adequate absorption process allowing biopeptides to cross the barrier and, thus, be available to contact with the HepG2 cells to predict hepatic metabolism (mimicking the availability of peptides in the blood stream and of its contact with hepatocytes in the liver) [18]. To increase the metabolic activity of liver and small intestine, an organ-to-organ interaction in vitro was created with a static simple double-layered system of Caco-2 and HepG2 cells separated by a semipermeable membrane culture insert [18,19]. After simulation of oral administration, the final BSG protein hydrolysate maintained their ACE-inhibitory potential (Figure 1 and Figure 2). However, when compared with captopril (an ACE-inhibitory drug), the final product improved its ACE-inhibitory ability by 3.7% (Table 1). Nevertheless, both initial and final BSG peptides exhibited a significantly greater effect than captopril (Table 1). On the other hand, BSY and MIX protein hydrolysates increased their ACE-inhibitory capability with simulated oral administration when compared with the control (Figure 1 and Figure 2). More importantly, although the initial BSY protein hydrolysate demonstrated a 26.8% lower effect in reducing ACE activity than captopril, the respective final product revealed a similar potency to the anti-hypertensive drug (Table 1). However, for MIX protein hydrolysate, the initial peptides unveiled a similar effect to captopril, which remarkably improved with simulated oral administration (Table 1). These results not only validate the oral route as an advantageous way to administrate these brewing protein hydrolysates, but also show, for the first time, that these peptides suffer changes during oral administration, either during gastrointestinal digestion, intestinal absorption, and/or metabolism, conditioning the therapeutic outcome. A possible explanation for this observation is that initial protein hydrolysates contain a mixture of peptides with extremely diversified length and composition, which could be further cleaved by gastrointestinal enzymes or by liver cells, forming new peptides able to inhibit ACE. This is supported by the broad range of possible substrates that ACE catalyzes, it not being surprising that several newly formed peptide sequences could lead to an inhibitory effect [34].

The evidence that the bioactivity of protein hydrolysates is influenced by oral administration is of extreme importance to raise awareness to researchers to consider the oral administration process before moving forward to animal studies, not only due to ethical issues (which are relevant and need to be taken into consideration) but also because we should formerly infer the adequate therapeutic dose range to be tested in vivo to avoid unnecessary studies. Indeed, most investigators rely solely on the concentrations that presented positive in vitro results without considering the impact of the oral route; however, the oral administration process might significantly influence and change the peptides, as our results demonstrated. In other words, if the oral route induces a decrease in the predicted effect, the in vivo analysis of that initial dose could result in the absence of effect since a higher dose could be necessary to achieve the expected outcome. By opposition, if the oral route increases the predicted effect, that initial dose could prompt hypotension or even adverse effects. Thus, following the present protocol of simulated oral administration, the dose can be adjusted accordingly.

We compared the final brewing protein hydrolysates with captopril (Table 1), the first ACE-inhibitory drug available on the market [5] which still used in clinical practice. Since the concentration of captopril used (1 µM) was more than 45-fold higher that its IC_50_ value [21], our results can be directly interpreted with an anti-hypertensive outcome.

BSY and BSG peptides also have reported antioxidant and anti-inflammatory capacities [7,15,16], which is of major importance since oxidative stress and inflammatory markers are correlated with high BP, and both pathological conditions are believed to be involved in the pathogenesis of primary hypertension [36,37]. In the current study these bioactivities were not explored, but if the reported antioxidant and anti-inflammatory effects are preserved after oral administration, it is expected that brewing protein hydrolysates will present multiple biological activities against the elevation of BP, having the possibility to provide more health benefits with a single bioactive component. These natural peptides could be extremely useful to lower BP as early onset of treatment, at the pre-hypertensive level, allowing the control of hypertension at an early stage of development and, thus, greatly decreasing the prevalence of hypertension and, consequently, CVD risk. Furthermore, these brewing protein hydrolysates could fill a market space that, for other types of indications, such as satiety, is already established by a product with a similar nature (protein hydrolysates from brewing yeasts) [38].

## 5. Conclusions

Altogether, the results gathered in the current study indicated that hydrolysates’ bioactivity can be influenced by oral administration. Indeed, the present brewing protein hydrolysates (BSY, BSG, and MIX) maintained or even enhanced their bioactivity as ACE-inhibitors after simulated oral administration. Furthermore, a novel MIX protein hydrolysate exhibited an exceptional efficiency in inhibiting ACE, revealing a 30% greater effect than a clinical antihypertensive drug, a similar result also being obtained for the final BSG peptides. Therefore, this work suggests BSG and MIX protein hydrolysates as safe brewing products with a great prospective of decrease BP and, thus, to pursue in order to develop natural anti-hypertensive products that could be marketed as food ingredients, supplements, nutraceuticals, or even drugs to reduce BP, filling the market space for this indication.

## Figures and Tables

**Table 1:** ACE-inhibitory capacity exhibited by brewing protein hydrolysates compared with an anti-hypertensive drug.

	ACE Activity of Protein Hydrolysates (as % of Captopril, 1 µM)	
	Initial	Final
BSY	126.8 ± 10.1 **	98.0 ± 5.6
MIX	113.4 ± 5.2	69.3 ± 7.2 **
BSG	81.4 ± 3.6 *	77.7 ± 5.2 **

Influence of natural peptides derived from brewing by-products on ACE activity presented as % of captopril. Brewer’s spent grain (BSG), brewer’s spent yeast (BSY), and 50:50 mixture of BSY:BSG (MIX) protein hydrolysates before (initial) and after (final) simulated oral administration were tested at 0.86 mg/mL (assuming all hydrolysates were absorbed by Caco-2 cells). Significant differences of ACE activity against captopril: * *p* < 0.05 and ** *p* < 0.01 (One-way ANOVA followed by post hoc Dunnett’s multiple comparisons *t*-test).

## Data Availability

The data presented in this study are available on request.
